# Mitochondrial Redox Regulations and Redox Biology of Mitochondria

**DOI:** 10.3390/antiox10121921

**Published:** 2021-11-29

**Authors:** Petr Ježek

**Affiliations:** Department of Mitochondrial Physiology, No.75, Institute of Physiology of the Czech Academy of Sciences, 14220 Prague, Czech Republic; jezek@biomed.cas.cz; Tel.: +420-296442760

Mitochondria undoubtedly represent a metabolic hub, but also act as a redox hub, controlling cell fate and emanating superoxide/H_2_O_2_, which in a regulated form and timing provide redox signaling. Retrograde redox signaling from the mitochondrion is directed towards targets located in the cell cytosol, nucleus, plasma membrane or other cell components. External redox signaling from the cell regulates components within the mitochondrion and may include the H_2_O_2_ activation of mitochondrial kinases. Finally, intra-mitochondrial redox signaling can be recognized.

Examples of verified acute retrograde redox signaling from mitochondrion are the inhibition of proline hydroxylase domain enzymes (PHD/EglN, also FIH), resulting in hypoxia-inducible factor-1α (HIF-1α) accumulation and transcriptome reprogramming; and fatty-acid- and branched-chain-ketoacid-stimulated insulin secretion, described in this Special Issue by an introductory review [1].

Note that studies on redox signaling represent the most dynamic part of the overall redox biology of mitochondria (reviewed in general in [2]). Redox pathogenic aspects rather stem from the excessive superoxide/H_2_O_2_ formation or from the insufficiency of the antioxidant mechanism (reviewed for a special case of pancreatic β-cells in [3]). The well-described intra-mitochondrial oxidative folding system is reported by Tokatlidis and colleagues [4], involving the Mia40 protein, which forms disulfide bridges on important proteins within the intermembrane space. The transcriptional regulation of mitochondrial redox equilibria is described by Scholtes and Giguère, presenting estrogen-related receptors as targetable redox sensors [5]. Jabůrek et al. [6] describe examples of both intramitochondrial redox signaling, as well as protein synergy leading to an antioxidant action. The former targets the H_2_O_2_-activated phospholipase iPLA2γ, relaying intramitochondrial redox signaling to free fatty acids as second messengers and simultaneously as cycling substrates of adenine nucleotide translocase and certain uncoupling protein isoforms. The latter phenomenon of fatty acid cycling results in a mild uncoupling of mitochondria, which effectively provides the attenuation of mitochondrial superoxide formation, unless mutated ND5 subunits of Complex I are present (or other subunits encoded by mitochondrial DNA are mutated).

This Special Issue is accompanied by a review illustrating the exceptional adaptability of mitochondria [7] and by three original articles [8,9,10], illustrating examples of redox biology, including mitochondria, and one article where bioenergetics aspects prevailed [11]. Dominiak and Jarmuszkiewicz [8] analyzed a possible maximum strength of redox signaling, considering a tissue-specific maximum respiratory chain capacity to produce mitochondrial reactive oxygen species (ROS) and proposed a new parameter RCR_ROS_, the ratio between the formation of mitochondrial ROS under nonphosphorylating and phosphorylating conditions. This reflects the maximum ROS increase when all ADP is phosphorylated. An exemplar study is presented by Drabik et al. [9], involving also retrograde redox signaling from the mitochondria to the nucleus in fibroblasts from patients diagnosed with the sporadic form of Alzheimer’s disease. A pro-oxidant, mitochondria-targeted drug FRI-1, inducing apoptosis of breast cancer cell lines is described by Córdova-Delgado et al. [10].
